# The impact of small-scale green infrastructure on the affective wellbeing associated with urban sites

**DOI:** 10.1038/s41598-023-35804-2

**Published:** 2023-06-15

**Authors:** Pablo Navarrete-Hernandez, Kate Laffan

**Affiliations:** 1 Department of Landscape Architecture, University of Sheffield, Western Bank, Sheffield, S10 2TN UK; 2 Department of Psychological and Behavioural Science, The London School of Economics, Houghton Street, London, WC2A 2AE UK

**Keywords:** Psychology, Environmental social sciences

## Abstract

The largest public space in any city is its streets. Investments which incorporate small-scale green infrastructure into streetscapes can bring more nature into the lives of urban residents worldwide, including those living in even the most economically and spatially constraint places. However, little is known about the impact of such small-scale investments on urban residents’ affective perceptions of their local environments and how to design these investments to maximise their positive impacts. In the current study, we use photo simulation techniques and an adapted form of the Positive and Negative Affective Schedule to examine the impact of small-scale green infrastructure interventions on the affective perceptions of low, middle and high-income sites in Santiago Chile. Our results, based on 62,478 reports of affective perceptions from 3,472 people, indicate that green infrastructure investments can both promote positive affect and, to a lesser, but still substantial extent reduce negative affect. The magnitudes of these relationships vary across discrete affective measures and for many of these measures, both positive and negative, a minimum of 16% increase in green coverage is required to see an impact. Finally, we find people associated lower affect with low, compared to middle and high, income sites but that these affective inequalities can be addressed, at least in part, through green infrastructure interventions.

## Introduction

The world’s urban population is expected to increase by approximately 1.5 times in the coming two decades-from 4.4 billion in 2023 to over 6 billion by 2045^1^. While urban living offers many social and economic benefits, it has also been linked to lower subjective wellbeing and mental health problems^2,3^. Trends towards increased urbanisation around the world beg the question: how can policymakers and planners promote the day-to-day wellbeing of people living in populated urban centres? Subjective wellbeing (SWB) measures, individuals’ reports of how they evaluate their lives and feel day to day, can help provide some insights^4^. One intervention for which there is a growing body of evidence in the subjective wellbeing literature is the provision of urban green infrastructure, including street trees, playing fields, parks and other open green spaces. Research indicates that urban residents report higher happiness, and employees report higher levels of workplace happiness, when living and working in settings with accessible green space^5–7^. Other work finds that green space in deprived urban areas is negatively associated with residents’ stress levels^8^, and that living in proximity to greenspace acts as a buffer to stressful live events^9,10^.

While this work highlights green infrastructure investment as a promising policy tool with which to enhance urban residents’ daily lives, it has tended to focus on a narrow range of measures of affective SWB, typically relating to happiness and stress. This focus precludes a nuanced understanding of the ways that green infrastructure affects urban residents’ day to day SWB. In a meta-analysis on the impact of spending time in natural environments compared to urban/built ones, McMahan & Estes^11^ find that natural environments are associated with a moderate increase in positive affect and a smaller, yet consistent, decrease in negative affect. It is unclear whether these findings will hold when the natural elements are incorporated into the urban environments, as well as the extent to which they will vary across different types of positive and negative affect. In other words, a comprehensive examination of the discrete emotional responses to urban green infrastructure is missing from the literature.

Furthermore, the work which has examined the relationship between urban green infrastructure and experienced SWB has tended to examine large-scale infrastructure such as public parks^3^. However, in populated areas within cities, public land and investment capacity is often limited^8–10^. Small-scale green infrastructure investments like street trees have lower space and money costs than do larger scale ones like public parks and are often more feasible and accessible. While such investment may not yield the same frequency of intention interactions with nature (i.e., visits with the specific goal of interacting), they likely create the conditions for many more incidental interactions in which people engage with nature while doing other things or while on route to doing other things (e.g. on route to work) and indirect interactions like viewing nature through the window^12,13^. Importantly too, small scale investments are also understood to pose lower risks of ‘green gentrification’ – a concern that has been raised in response to the greening of low-income areas^14^. Examining the impact of small-scale green infrastructure can help inform strategies to make urban areas ‘just green enough’ to increase the supply of green infrastructure and yield the associated wellbeing benefits for residents in disadvantaged communities while incurring the lowest possible opportunity costs^15^.

Relatedly, understanding how the extent and content of such small-scale infrastructure investments – for example the amount and type of street vegetation – influences their impact can help further inform investment decisions. In their examination of the health impacts of urban nature, Shanahan and colleagues^16^ put forward a nature-dose framework in which suggest that the impact of nature depends on the intensity (the quality and quantity of nature), as well as the frequency and duration of the exposure to it. Empirical work that has built on this framework finds that while vegetation complexity—an aspect of nature quality—is not predictive of mental health problems^17^, quantity of vegetation cover is negatively so^12^. More specifically, this latter work finds evidence of a threshold response at with the population prevalence of mental health issues is significantly lower beyond a minimum threshold of nature coverage^12^. Other work has emphasised the importance of going beyond categorising green space as ‘simply green’ to examine different types of greenspace like urban green space compared to agricultural land, and demonstrated that associations with mental health vary across these and other types^18,19^. Together this work highlights the importance of understanding the intensity of nature required (both in terms of quality and quantity) to enhance urban wellbeing. Cox and colleagues^12^ also identified afternoon bird abundances as a key feature of local nature which was negatively associated with mental health problems and Carrus and colleagues^20^ find that the richness of biodiversity in urban green areas moderates their impact on subjective wellbeing. This finding suggest that the benefits of urban green infrastructure arise not only from the green itself but also from the biodiversity it supports.

Lastly, in many urban areas around the world, access to and quality of green infrastructure varies across socio-economic groups, with disadvantaged communities tending to reside in areas with poorer environmental quality^21,22^. Although research into the impact of green infrastructure of people’s perceptions of the safety of urban green space has been shown to vary across the socio-economic profile of the respondents^23^, to date no work has considered whether green infrastructure yields equivalent experienced SWB benefits in low, compared to medium or high, income neighbourhoods. Understanding the relationships between SWB and green infrastructure across neighbourhoods with different socioeconomic profiles can help to shed light on the inequalities in lived experiences of those living in these different places, as well as the potential of targeted green infrastructure to reduce such inequalities.

In the current work, we explore the impact of green infrastructure on the affective perceptions different urban spaces using experimental methods, photo simulation and measures derived from the Positive and Negative Affect Schedule (PANAS)^24^. While exposure to photos is of course not the same as full immersion in the urban environment, existing studies indicate that people’s mood and stress biomarkers are positively impacted by viewing images of nature^25,26^ and neuroscientific evidence indicates that viewing natural images also results in the activation of brain areas associated with affect such as the amygdala and the putamen^27^. Given that the current study aims to explore the impact of green infrastructure investments on perceptions of urban sites prior to their being implemented, it is necessary to rely on simulations of nature. These simulations can either come in the form of photographs or as immersive virtual reality environments. Both approaches have been shown to impact on wellbeing related outcomes, with some recent work indicating that photographs are more effective that simulated immersive environments at reducing physiological arousal^27^.

**Figure 1:**
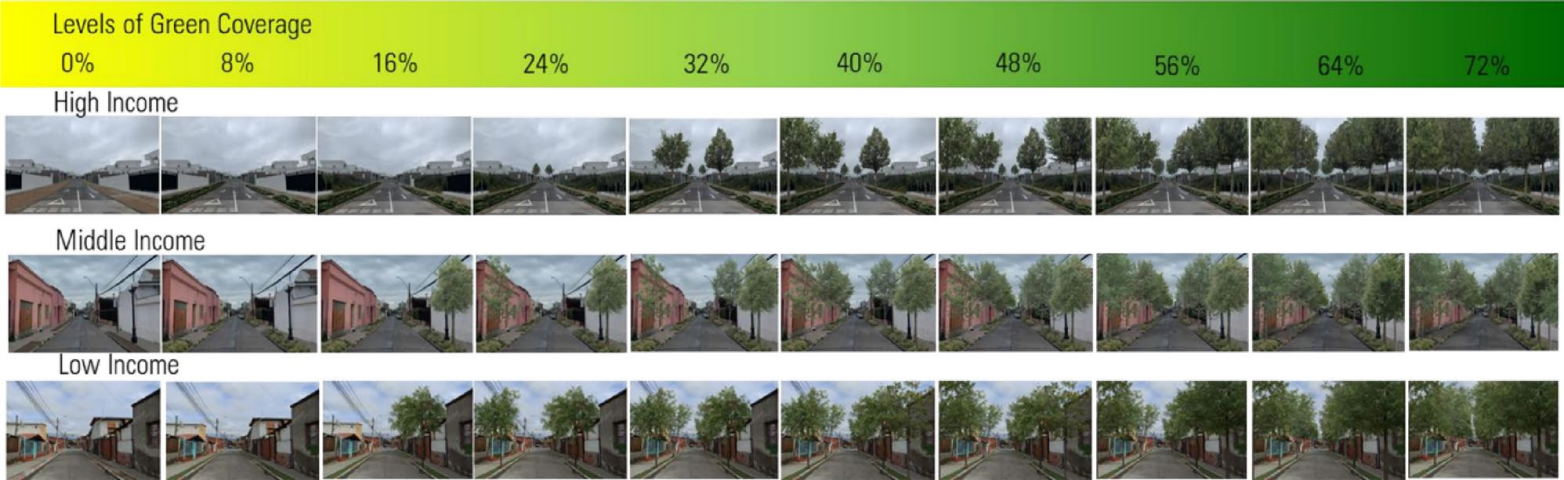
Simulated images from low, middle and high income areas across different levels of green coverage.

In our study, we presented 3,472 participants with multiple images of urban sites on an online platform – www.urban-experiment.com – and asked them to rate the perceived affect associated with the sites according to one randomly chosen measure from the 20 items in the PANAS scale. Each participant rated 18 images in total such that we collected 62,478 images ratings. The images consist of real urban sites from across the city of Santiago in Chile and vary in terms of the income profile of the neighbourhoods they represent, as well as the amount of green infrastructure that they include. As such, we are able to examine the impact of increases in green infrastructure ranging between 0–72% on people’s perceptions of low, middle and high income urban sites on composite indices of positive affect (PA) and negative affect (NA), as well as across 20 different types of affect. See Fig. 1 for example images.

The results indicate that positive affect is increasing with green coverage: on average an 8% rise in green coverage in the photo is associated with a 0.230 increase on a 1–10 point scale. When we disaggregate the results according to the discrete emotions, we find that green coverage also positively impacts nine of the ten positive emotions covered by the PANAS scale, with green coverage being most closely associated with enthusiasm, pride and excitement. The exception is alertness which we find is significantly negatively impacted by increases in green space coverage. Looking across the distribution of increases in green space coverage the relationship with the PA index appears to be linear, with no obvious evidence of diminishing returns to increased coverage. This pattern largely holds across the different PA measures, however, there is evidence that the impacts of green coverage only kick in at 16% coverage or greater for many of the individual PA measures and there is also some evidence of levelling off at relatively high levels of green coverage in the cases of feeling active, proud and attentive.

We also find a negative impact of green space coverage and negative affect. This impact is smaller in magnitude than the impact on positive affect with the average reduction in negative affect brought about by an 8% increase in green coverage being 0.143 on a 1–10 scale. Green coverage increases lead to significant decreases in all ten measures of negative affect, with the greatest reductions pertaining to reports of irritability and upset. The relationship between green coverage and negative affect is linear up until about 56% green coverage, after which there are no further reductions with increased green space coverage. The individual measures of NA follow a similar pattern, though many are only negatively impacted at green coverage levels of 16% or greater.

Turning finally to the income heterogeneity analysis, images of low-income neighbourhoods are associated with lower PA and higher NA than are medium- and high-income neighbourhoods, highlighting affective inequalities between these areas. Additionally, although PA perceptions of images of these three different types of neighbourhoods are positively impacted by increases in green infrastructure, the magnitude of the average impact in higher in high-income neighbourhoods relative to low and middle income sites. When looking at NA overall, the decreased negative affect associated with increased green space coverage is of an equivalent magnitude in response to images of low-income neighbourhoods compared to medium- and high-income ones.

Overall, the results suggest that green infrastructure investments can both promote PA and to a lesser extent reduce NA, underlining the potential subjective wellbeing benefits of such interventions. Importantly, however, for many of the discrete affect measures, both positive and negative, a minimum of 16% increase or greater is required to see an impact. Furthermore, given the worse affect ratings associated with sites in low-income areas and the relationships between affect and green space coverage in areas with low-income profiles, green infrastructure investments represent a promising intervention to enhance the day to day lives of those living in low-income neighbourhoods and narrow inequalities in affective perceptions of urban areas.

## Results

### Green coverage and positive affect

**Figure 2:**
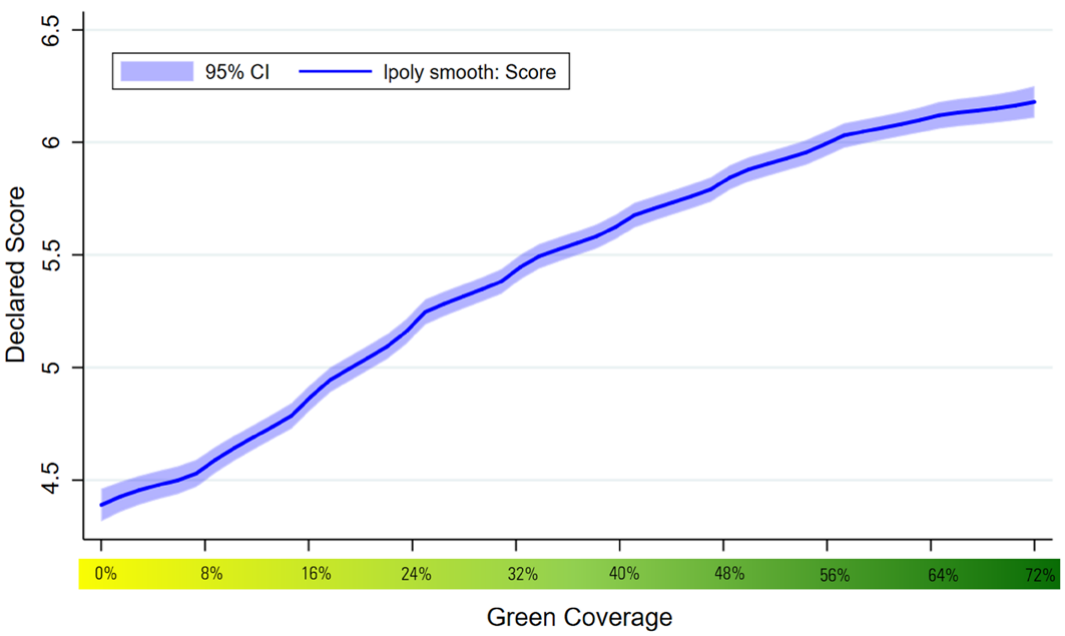
The impact of green coverage on the positive affect index. *Note*. The graph reflects 20,786 PA ratings from 2,335 people. Mixed regression estimates with and without controls are very consistent and can be found in the Supplementary Material in Table A2. The reader should note that the y-axis shows a small section of the 1 to 10 scale range available to participants.

**Figure 3:**
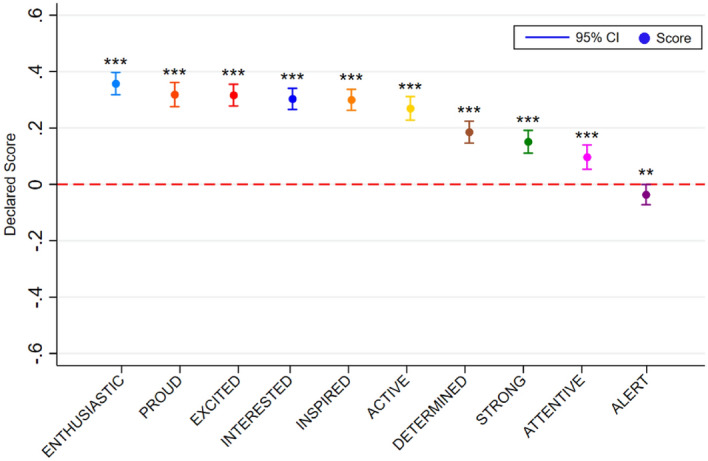
The average impact of an 8% increase in green coverage on measures of positive affect. *Note*. The graph reflects 20,786 PA ratings from 2,335 people. Using different subsamples for each affect, this figure reports OLS regressions results of average deviations of a selected affect with an 8% increase in street green coverage. Estimates without controls are shown. Each point represents the average treatment effect compared with images with no green intervention. Mixed regression estimates with and without controls are very consistent and can be found in the Supplementary Material in Table A3 and A4. The reader should note that the y-axis shows a small section of the 1 to 10 scale range available to participants. Capped spikes represent 95% confidence intervals. *P < 0.1, **P < 0.05, ***P < 0.01.

**Figure 4:**
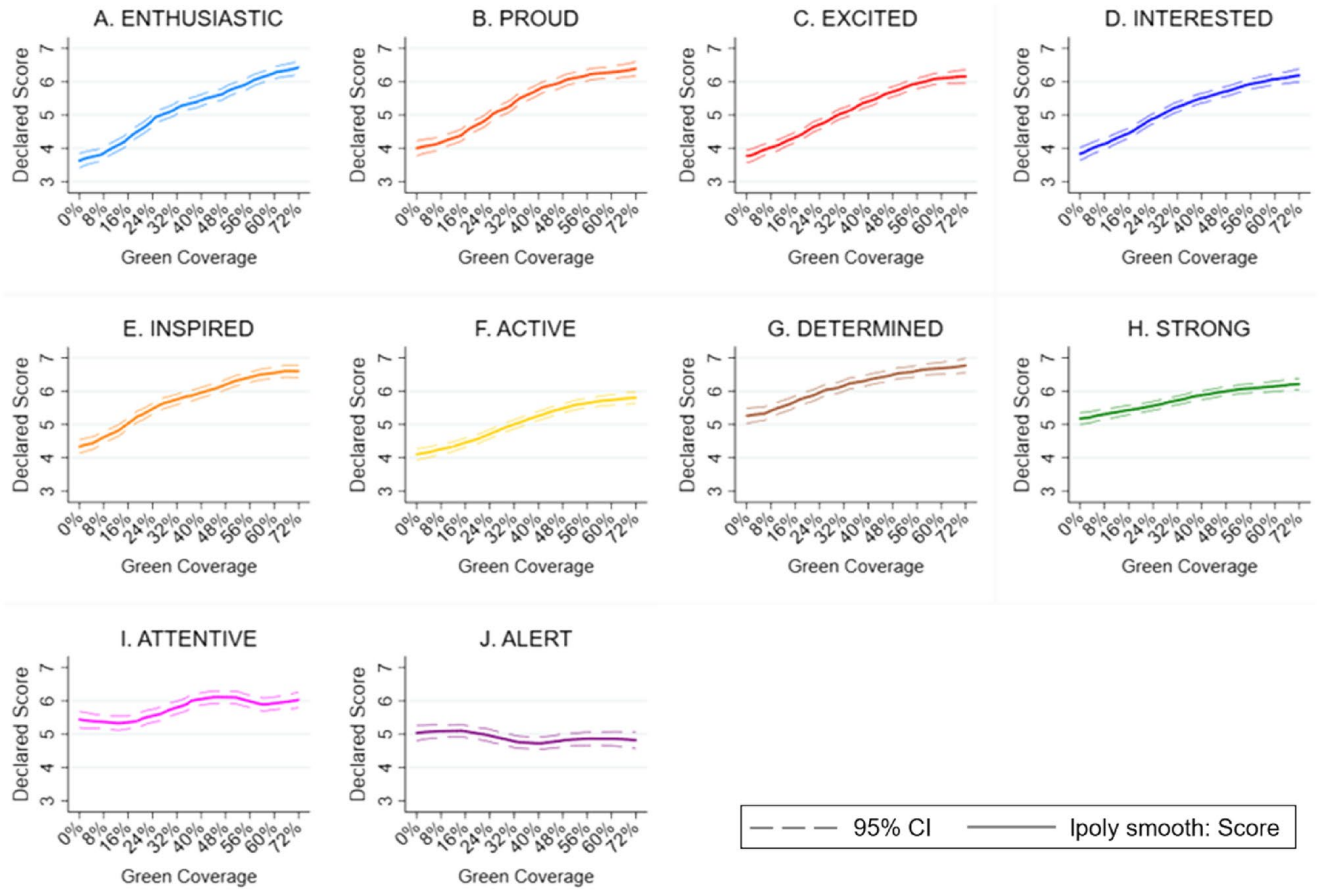
The impact of green coverage on the discrete positive affect measures. *Note*. Together the graphs reflect 20,786 PA ratings from 2,335 people. Estimates without controls are shown. Mixed regression estimates with and without controls are very consistent and can be found in the Supplementary Material in Table A5 and A6. The reader should note that the y-axis shows a small section of the 1 to 10 scale range available to participants.

Increases in green infrastructure coverage positively impact the PA index, both when controls (gender, age, education, income, country of residence, allergy status and frequency of visits to parks) are excluded and included. See Table 1. On average an 8% increase is associated with 0.23 higher reports of PA. This relationship is broadly linear across the range of different levels of green space coverage (see Fig. 2). Of the discrete PA measures, all but one of the measures is significantly positively impacted by increases in green coverage (interested, excited, strong, inspired enthusiastic, determined, attentive, active and proud, but not alert). The greatest increases relate to enthusiasm, pride and excitement (see Fig. 3). The smallest increases are related to strong and attentiveness and there is evidence of a negative relationship between increased green coverage and alertness. These relationships are predominantly linear but for a majority of the measures – excited, strong, alert, enthusiastic, determined, active, proud – the positive impact only applies once green coverage is at least 16% (i.e., there is no significant impact of the change from 0–8% green coverage) (see Fig. 4). There is also evidence of further non-linearities in the cases of active, pride and attentiveness.

**Table 1:** The impact of green coverage increases and PA index.

Variables	Positive affect	
	1	2
Green coverage (+ 8%)	0.229*** (0.00690)	0.230*** (0.00679)
Constant	4.153*** (0.0497)	3.996*** (0.250)
Controls (1)	No	Yes
Observations	20,786	20,786
Number of groups	2,335	2,335

We use the sample of all participant street image ratings responding to 10 positive affect questions. This table displays OLS regressions results of average change in negative emotions resulting from an 8% increase in street green coverage. The results are estimated running Eq. (1) on the responses to the positive affect questions. Mixed regression estimates without and with controls are presented in column 1 and 2 respectively. Control variables include all the observed participant’s characteristics: gender, age, educational level, income, country of residence, allergies to plants, pollen or insects, and frequency of visiting a park and the date of the survey.

Robust standard errors in parentheses.

*P < 0.1, **P < 0.05, ***P < 0.01.

### Green coverage and negative affect

**Figure 5:**
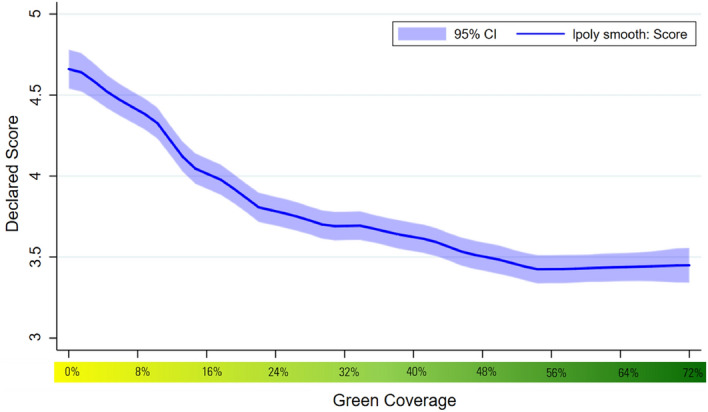
The impact of green coverage on the negative affect index. *Note*. The graph reflects 9,716 NA ratings from 1,105 people. Estimates without controls are shown. Mixed regression estimates with and without controls are very consistent and can be found in the Supplementary Material in Table A2. The reader should note that the y-axis shows a small section of the 1 to 10 scale range available to participants.

**Figure 6:**
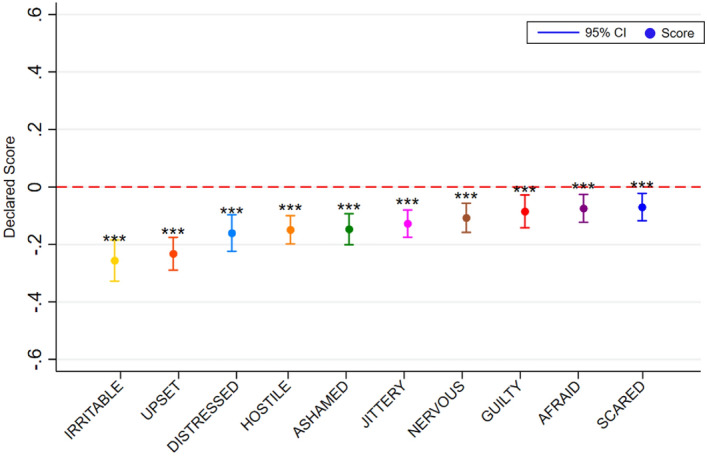
The average impact of an 8% increase in green coverage on measures of negative affect. *Note*. The figure reflects 9,716 NA ratings from 1,105 people Estimates without controls shown. Using different subsamples for each affect, this figure reports OLS regressions results of average deviations of a selected affect with an 8% increase in street green coverage. Estimates without controls are shown. Each point represents the average treatment effect compared with images with no green intervention. Mixed regression estimates with and without controls are very consistent and can be found in the Supplementary Material in Table A7 and A8. The reader should note that the y-axis shows a small section of the 1 to 10 scale range available to participants. Capped spikes represent 95% confidence intervals. *P < 0.1, **P < 0.05, ***P < 0.01.

**Figure 7:**
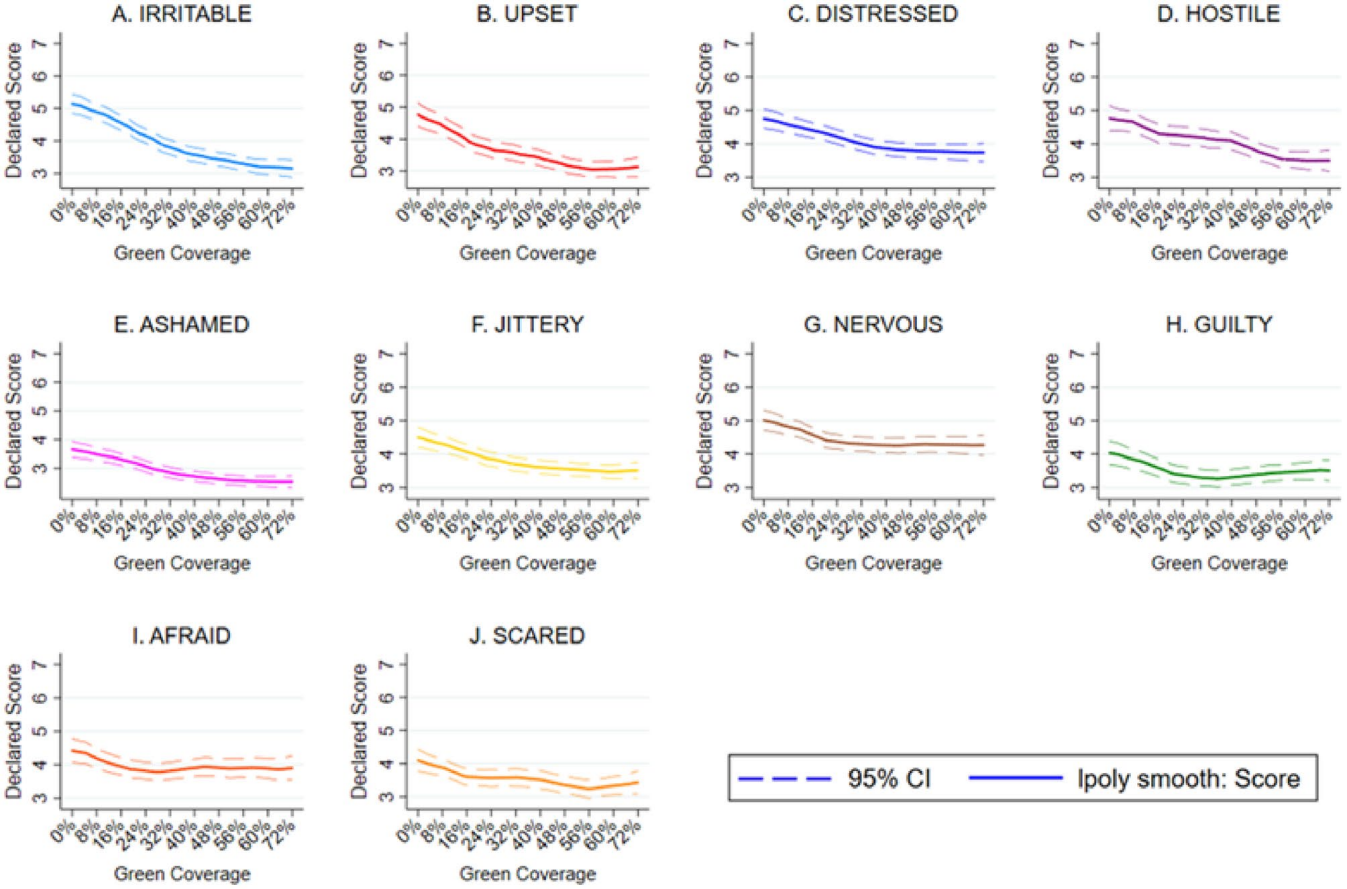
The impact of green coverage on the discrete negative affect measures. *Note*. Together the graphs reflect 9,716 NA ratings from 1,105 people. Estimates without controls shown. Mixed regression estimates with and without controls are very consistent and can be found in the Supplementary Material in Table A9 and A10. The reader should note that the y-axis shows a small section of the 1 to 10 scale range available to participants.

Although green coverage negatively impacts on NA both when controls are and are not included, the effect is smaller in magnitude compared to PA: on average an 8% increase is associated with 0.14 lower reports of NA (see Table 2). Additionally, this relationship is not as clearly linear. At low levels of green space coverage additional green has a relatively larger negative impact than at higher points in the range. After 56% green coverage there are no further returns to increased green coverage (see Fig. 5). All ten discrete NA measures are negatively impacted by increases in green space coverage (distressed, upset, guilty, scared, hostile, irritable, nervous, ashamed, jittery and afraid) (see Fig. 6). Of the measures, the magni^1^tude of reductions is greatest for irritability and upset and smallest for scared, afraid and guilt. Echoing the relationship between green coverage and NA overall, the relationships with specific NA measures are linear over relatively small increases in green coverage but level off at higher levels. As was the case with PA, many of the significant negative impacts on NA only appear after the green space coverage is increased by at least 16% or more, i.e., those relating to feeling distress, guilt, scared, hostile, irritable, nervous and afraid (see Fig. 7).

**Table 2:** The impact of green coverage increases and NA index.

Variables	Negative affect	
	1	2
Green coverage (+ 8%)	− 0.143*** (0.00911)	− 0.143*** (0.00899)
Constant	4.597*** (0.0745)	5.245*** (0.444)
Controls(1)	No	Yes
Observations	9,716	9,716
Number of groups	1,105	1,105

Robust standard errors in parentheses.

*P < 0.1, **P < 0.05, ***P < 0.01.

We use the sample of all participant street image ratings responding to 10 negative affect questions. This table displays OLS regressions results of average deviations of negative emotions with an 8% increase in street green coverage. The results are estimated running Eq. (1) on the sample of all street images responses to negative affect questions. Mixed regression estimates without and with controls are presented in column 1 and 2 respectively. Control variables include all the observed participant’s characteristics: gender, age, educational level, income, country of residence, allergies to plants or insects, frequency of visiting a park and the date of the survey.

### Green coverage and affect: Heterogeneity across neighbourhood income profiles

**Figure 8:**
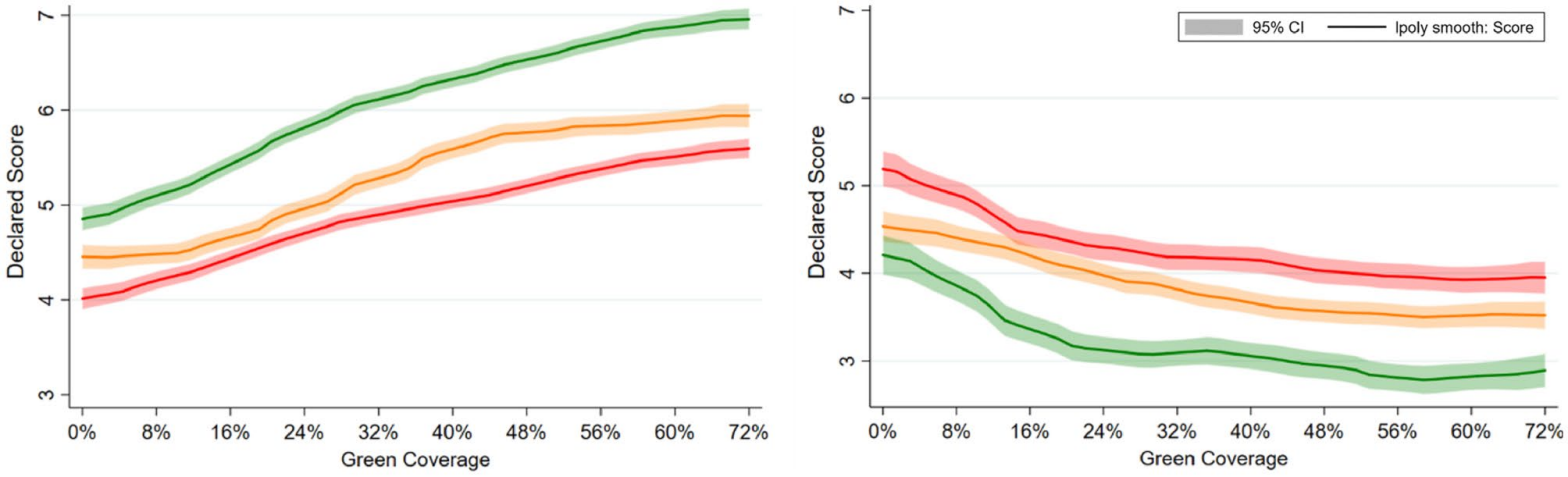
The impact of green coverage on the PA and NA affect indices by neighbourhood income profile. *Note*. Together the graphs reflect 786 PA ratings from 2,335 people and 9,716 NA ratings from 1,105 people. Estimates without controls are shown. Mixed regression estimates with and without controls are very consistent and can be found in the Supplementary Material in Table A11. The reader should note that the y-axis shows a small section of the 1 to 10 scale range available to participants.

When we disaggregate the affective perceptions of low-, middle- and high-income urban sites we find large variation in both PA and NA, with low-income sites being consistently ranked lowest in PA and highest in NA followed by middle- and then high-income sites. Turning then to the differences in green coverage impacts in areas with different income profiles we see that, the impact of green coverage on positive affect is relatively smaller for images of low- and middle-income areas compared to high-income areas and that all three relationships are largely linear across the different green coverage distribution. Green coverage has similar negative impacts on the NA index across all three area types, with the impacts being concentrated at the lower end of the green coverage distribution in all three cases. See Table 3 and Fig. 8.

**Table 3:** Green coverage interacted with neighbourhood income levels and PA and NA.

Variables	Positive affect		Negative affect	
	1	2	3	4
Green Coverage (+ 8%)	0.212*** (0.00950)	0.212*** (0.00957)	− 0.143*** (0.0135)	− 0.146*** (0.0137)
Middle Income	0.470*** (0.0693)	0.464*** (0.0698)	− 0.473*** (0.102)	− 0.505*** (0.102)
High Income	0.805*** (0.0901)	0.789*** (0.0910)	− 1.058*** (0.131)	− 1.072*** (0.133)
Middle Income*Green Coverage (+ 8%)	− 0.0141 (0.0108)	− 0.0133 (0.0109)	0.00659 (0.0160)	0.0117 (0.0161)
High Income*Green Coverage (+ 8%)	0.0666*** (0.0124)	0.0683*** (0.0125)	− 0.00520 (0.0182)	− 0.00326 (0.0185)
Constant	3.726*** (0.0678)	4.127*** (0.254)	5.106*** (0.100)	5.263*** (0.473)
Controls	No	Yes	No	Yes
Observations	20,786	20,458	9,716	9,353
Number of groups	2,335	2,297	1,105	1,063

Robust standard errors in parentheses.

*P < 0.1, **P < 0.05, ***P < 0.01.

## Discussion

Green infrastructure represents a promising policy tool with which to enhance the daily lives of people living in urban areas. In the current work, we add to the evidence in support of this demonstrating that green infrastructure positively impacts the perceptions of urban sites in Santiago Chile across a wide range of positive and negative affective measures. While existing work has compared the affect derived from natural versus urban sites^11^, and separately looked at the association between large scale urban nature and wellbeing^28^, our work looks specifically at incorporating small-scale natural elements into urban sites. This kind of green infrastructure costs less to put in place and likely creates more opportunities for incidental and indirect nature engagement than large urban parks^12^. The results indicate that such interventions can significantly enhance the affective perception of urban sites, with particularly strong impacts on positive relative to negative affective measures. The findings build on previous work that has identified negative associations between small-scale green infrastructure and mental health problems^29^ and feelings of stress^30–32^, suggesting that it can promote positive affect as well as protecting from negative states.

The work’s findings also provide richer insights than have been available to date into the relationship between green space coverage and a large range of discrete affective perceptions. While there are largely consistent patterns between the specific affect measures and green coverage – with nine of the ten PA measures being significantly positively impacted by increases in green coverage and ten of the ten NA ones being negatively so – the magnitudes of the relationships vary substantially. This is of importance if there are specific affective perceptions of interest to an urban planner. For example, imagine an urban planner is interested in impacting feelings of pride associated with an urban site. Our results highlight the strong impact of green infrastructure investments on such feelings. This may be related to the enhanced feelings of social cohesion and place identity that have been linked to green infrastructure^33,34^. At the same time if the planners focus is on reducing feelings of fear, our results suggest that although such perceptions of urban sites are significantly reduced by increases in green coverage the impact is smaller than on other negative affect measures like feelings of irritability and upset. Maas and colleagues^35^ find that urban green space is associated with enhanced feelings of safety except in very densely populated areas, where the relationship turns negative. These findings likely explain at least in part the overall weaker relationship with feelings of fear documented in this work. Though this is the first work of its kind to look at the relationship between green infrastructure and all the emotions in the PANAS scale, other work using social media data has begun to provide insights into the prevalence of specific emotions in green space. For example, Zhu and colleagues^36^ use facial expression detection, and Roberts and colleagues^37^ use sentiment analysis of Twitter data, investigating links between emotions like happiness, surprise, anger, fear and anger, sadness and disgust and green coverage in the authors’ vicinity. Further work using a range of different methodologies is needed to expand the literature in this area. When looking to influence the affective experiences of urban communities through green infrastructure investments, it is important that urban planners are clear about the type(s) of affect they aim to target.

Our study also provides insights into the importance of the magnitude of green space coverage necessary to achieve changes to affective perceptions. While the PA and NA indices are associated with positive and negative average impacts respectively, the relationship is linear across the green coverage distribution for PA but tapers off after 52% coverage for NA. Given the costs associated with greater and greater levels of green infrastructure and assuming both enhancing PA and reducing NA are important to urban planners a medium scale investment (up to 52%) may represent an attractive investment. Additionally, when looking at the discrete affect measures, relating to both PA and NA, many of the impacts occur at 16% or greater green space coverage. Assuming that these discrete emotions are important to planners, these results suggest for green infrastructure to yield a range of affective benefits it also needs to be sizeable enough (+ 16% or greater). These findings echo the threshold effects documented elsewhere in relation to the health impacts of nature. For example, Cox and colleagues ^12^, find the lower thresholds for the mental health benefits of nature to be in the range of 20–30%, while Jiang finds optimal stress reduction at green coverage levels of 41% when measured looking at panoramic views and 20% when measures via satellite images. While identifying an exact minimum threshold requires further work and may vary across contexts, together these findings suggest that for green infrastructure to yield many of its affective benefits there needs to be enough of it.

Finally, the results highlight both stark differences in people’s’ affective perceptions of low- middle and high-income urban sites. These results suggest that urban residents’ daily affective experiences are likely influenced by their local environments, with those living in low-income areas suffering from disadvantages in both income and affect. However, we also find consistent relationships between affective perceptions and increases in green infrastructure in these places. Taken together these two findings suggest that the inequalities in experienced wellbeing of those living in urban sites with different income profiles could be addressed, at least in part, through street-level green infrastructure investments. This is particularly important to emphasise given the wealth of evidence of inequalities in access to green infrastructure in many urban areas around the world^20,21^.

While the study’s findings pertain to urban sites in Santiago Chile, the method used to explore the impact of green infrastructure on affective perceptions is broadly applicable to urban sites worldwide. Future research should look to examine the consistency of these findings in different geographical areas as well as contrast them with the impacts from other urban interventions options, such as street art or the inclusion of historical monuments^38^. Future work should also go beyond examining the quantity of green included in an investment to varying the type and vegetation complexity as well as other features of biodiversity like the inclusion of colourful flowers^39^ and nature congruent olfactory and auditory stimuli^30^ to further shed light on the impact of nature intensity on positive and negative affect^16^.

This study is not without limitation, opening opportunities for new research. First, we ran this experiment in a non-representative sample, i.e., while this research has a strong internal validity, further research needs to be carried out to show its external validity in other populations and geographies. The study is also only considers affective perceptions, excluded other wellbeing related perceptions including those related to feelings of meaning and connection^13^. We incorporate green space dominated by bushes and perennial trees in a cloudy winter season. The results may vary if other vegetation specifies were incorporated or other seasons were considered^40^. A final limitation is the use of static visual images. More immersive and dynamic techniques, like 360° videos and virtual reality for example, could lead to different results. While, there is no a priori reason to expect that the effects would change in direction, we can expect that the magnitude of impacts might be greater if delivered using these techniques^41^. This might be especially the case when efforts are made to to recreate a multisensory experience by including important factors like auditory sounds and smells^30,43^. Further avenues for research could explore how each of these aspects influence emotions in street-level green infrastructure.

Overall, the results of this study emphasise the potential of green infrastructure to enhance the affective perceptions of urban sites, while also speaking to urban planning considerations in terms of both the size and distribution of green infrastructure.

## Methods

### Experimental design

To assess the relationship between of green infrastructure and affect we conducted an online experiment involving photo simulation and an adapted form of the PANAS scale. We collected the data for this study between October 16th and November 18^th^ 2021. The research was approved by the Universidad de Chile, Faculty of Architecture and Urban Studies review board protocol 0123. The study took an average of 3:37 min and we did not provide incentives to participate in the study. Once people agreed to participate, they completed an online registration process which included a questionnaire about their socio-demographic information and attitudes towards urban nature, a brief about the study protocol and an informed consent form. After this, participants reported on their affective perceptions of 18 images of urban sites. Of those that signed the informed consent form online, 96.6% completed the survey.

We recruited a sample of 3,472 participants to participate in this study. To do so we asked university students enrolled in a course on the built environment and emotion to post a standardised advertisement for participants on their social media profiles on both Facebook and Instagram inviting them to complete a University of Chile survey on their feeling in public spaces in Santiago de Chile. The vast majority of the sample were Chilean residents (96.7%) and the sample was biased towards being female (64.5%) and younger than the Chilean population as a whole (64.5% were under 30). See Table A1 of the Appendix. The resulting sample is of 62,478 images ratings from 3,472 participants number of respondents.

To ensure participants characteristics were balanced across affect measures and levels of green intervention, we use an automated double randomization process. First, we randomly assigned participants a unique the order of appearance of the 18 street images levels and a randomly selected measure of affect. In doing this, we minimised the impact of order effects for any particular site and managed to cover all 20 affect measures in the PANAS scale. After this, for 9 of the images, one of the ten levels of greenery ranging over (0–72%) for each street was selected at random. The other 9 image acted as placebo images and did not include any intervention. As a result, a unique combination of order of appearance, intervention level and affect measure was assigned to each participant, allowing for balanced control and treatment groups^19^.

### Photo simulation

We used a collection of images to reflect the low-, middle- and high-income neighbourhoods. To select the study images, we first classified municipalities in Santiago into three socioeconomic groups based on data from the Chilean Ministry of Social Development^44^ and photographed nine different sites that reflected the three types of area. To obtain comparable street images, all photographs were taken at 1.7 m from the ground at the centre of a street which included residential buildings of one or two stories, narrow sidewalks (1.5–2 m) and two car lanes. We then ran a short pilot survey where people classified our collection of images as belonging to either low, middle or a high-income neighbourhood. Based on this pilot, for use in the final study, we chose those images that achieved a high level of correct classification (above 80%). We selected 9 images from which we removed all green elements to create our control images. Then through a series of computerised visual simulations, we incorporated different scales of street-level of green infrastructure, such as street trees and shrubbery, to create our treatment images. The percentage increase in green coverage in the images reflects the changes in the pixels from not green to green resulting from the edits and ranging between 8–72% at intervals of 8% increases. The editing was carried out in Photoshop by an expert in photo editing. See Fig. 1 for three examples of edited photos. We additionally incorporated 9 photographs with no intervention as placebos which were selected from the highest correctly classified scores from each municipality but not selected for green interventions.

### The PANAS scale

The PANAS assesses both PA and NA using a validated 20-item instrument^24^. The 10 items associated with positive affect relate to feeling: interested, excited, strong, inspired, alert enthusiastic determined, attentive, active and proud. The 10 items associated with negative affect relate to feeling: distressed, upset, guilty scared, hostile, irritable, nervous, ashamed, jittery and afraid. One of the advantages of PANAS is its simplicity, validity and reliability across different time scales and populations. The scale can be used to examine the higher order constructs of PA and NA, as well as specific feelings and emotion using the individual items, while still preserving its validity^24,45^. In this study we ask respondents to imagine they were walking in the urban site shown in the picture and to report on how much of a given type of affect they would feel in this public space on a 1–10 scale from very slightly/not at all to extremely. These questions are all posed in Spanish. We combine responses to these questions into separate PA and NA indices by taking the average of the ten positive measures and ten negative ones, as well as looking at responses to each affect measure in isolation.

### Statistical models

We collect participant reports of their perceptions of the 20 different affect measures associated with the urban sites. The main outcomes of interest in this study are the two composite affect indices, as well as the 20 discrete affect variables. In the analysis, we use random intercept models with fixed effects at the image level to explore the relationship between percentage of green infrastructure included and affect. We include the random intercept to account for the fact that each respondent reports on their perceptions of 18 different images, and as a result, these reports are not independent of one another. We include the image fixed effects to control for each image’s average affect ratings. Equation 1a takes the following form:

1 $${\text{Perception}}_{{{\text{i}},{\text{j}}}} = \beta_{{1}} {\text{Treatment\_Continuous}}_{{\text{i}}} + \beta_{{2}} {\text{Image}}_{{\text{i}}} + \beta_{{3}} {\text{Z}}_{{\text{i}}} + {\text{U}}_{{\text{j}}} + {\text{E}}_{{{\text{i}},{\text{j}}}}$$

where Perception*ij* is the perception rating given on a scale of 1 to 10 to the *i*th image by the *j*th individual. Treatment___Continuous is a continuous variable equal to zero if the *i*th image contains no green street intervention (control) or numbers one to nine each reflecting an 8% increase in green infrastructure coverage (treatments). Z represents a vector of control variables: the participants’ gender, age, educational level, income, country of residence, allergies to plants, insects or pollen, frequency of visiting a park and the date of the survey (if participant responded before or after November 4th). We included nature-based allergies to plants, insects or pollen as people suffering from such allergies might associate green infrastructure with negative affective experiences. We include frequency of park visits as an engagement with urban nature proxy for preferences for green spaces. Regarding the date of participation, we collected perceptions of positive affect first (October 16th and November 18th 2021) and then both positive and negative perceptions (November 4th-18th 2021). This strategy enabled us to hit our target sample size on positive emotion perceptions prior to incorporating negative affective perceptions into the study. We account for this in the analysis by including a control for the collection point. The coefficient of central interest to us is β1, the Average Treatment Effect, which captures the effect of different levels of green interventions on people’s reports of the affect they associate with the urban sites. Image i is an image fixed effect for the ith image included to control for the fact that each image has different average perception ratings, reflected by β2. Uj is the random intercept associated with the jth individual, and Eij is the error term. We report significance levels at 5% and 1% while considering a 5% a reasonable threshold for the avoidance of Type 1 errors.

We then estimate Eq. (2) which echoes Eq. (1) but treats green space coverage as a categorical variable equal to zero if the *i*th image contains no green street intervention (control) or numbers one to nine indicating that they contain 8%, 16%, 24%, 32%, 40%, 48%, 56%, 64%, 72% respectively, of images are covered by green infrastructure (treatments). We also generate affect/green infrastructure curves (y-axis for perceptions and x-axis for the quantity of green space) to graph the relationship between increases in green infrastructure and the range of affect outcomes. See Figs. 2, 4, 5 and 7.

2 $${\text{Perception}}_{{{\text{i}},{\text{j}}}} = \beta_{{1}} {\text{Treatment\_Categorical}}_{{\text{i}}} + \beta_{{2}} {\text{Image}}_{{\text{i}}} +_{{}} \beta_{{3}} {\text{Z}}_{{\text{i}}} + {\text{U}}_{{\text{j}}} + {\text{E}}_{{{\text{i}},{\text{j}}}}$$

Finally, in Eq. (3) we examine the relationship between green infrastructure and affect in low-, medium- and high-income sites. To do this, we interact the continuous treatment variable with a categorical variable indicating the type of neighbourhood. This model takes on the following form:

3 $${\text{Perception}}_{{{\text{i}},{\text{j}}}} = \beta_{{1}} {\text{Treatment\_Continuous}}_{{\text{i}}} + \, \beta_{{2}} {\text{Image}}_{{{\text{i }} + }} \beta_{{3}} {\text{Income}}_{{{\text{i }} + }} \beta_{{4}}$$

All existing notation is as before. Income is a categorical variable with three levels where low income represents the reference category. The coefficient of central interest to us is β4, which represents the interaction between the type of neighbourhood and the treatment. We also generate affect/green infrastructure curves for the three different types of neighbourhoods by running the EQ1 on the subsample of the neighbourhoods of different income types separately. See Fig. 8.

## Supplementary Information

Supplementary Information.

## Data Availability

The full data from this study is archived, discoverable and citable with metadata identifiable through a Digital Object Identifier reserved through the data repository Zenodo at the following (2015).
