# Supplementary material for: The impact of small-scale green infrastructure on the affective wellbeing associated with urban sites
Source: Sci Rep. 2023 Jun 15;13:9687. doi: 10.1038/s41598-023-35804-2 (PMC10272133; doi:10.1038/s41598-023-35804-2)
Supplement: Supplementary file 1 — Supplementary Information. [file 41598_2023_35804_MOESM1_ESM.docx]

## Supplementary material

Table A1. Descriptive statistics for the sample

| **Variable** | | | | **N** | **Percentage** |  |
| --- | --- | --- | --- | --- | --- | --- |
| Gender |  |  |  |  |  |  |
|  | Female |  |  | 40284 | 64,50% |  |
|  | Male |  |  | 21150 | 33,80% |  |
|  | Other |  |  | 1073 | 1,70% |  |
|  |  |  |  |  |  |  |
| Age |  |  |  |  |  |  |
|  | ≤30 |  |  | 40288 | 64,50% |  |
|  | 31-40 |  |  | 7761 | 12,40% |  |
|  | 41-50 |  |  | 7184 | 11,50% |  |
|  | ≥51 |  |  | 7274 | 11,60% |  |
|  |  |  |  |  |  |  |
| Have children | |  |  |  |  |  |
|  | No |  |  | 41674 | 66,70% |  |
|  | Yes |  |  | 20833 | 33,30% |  |
|  |  |  |  |  |  |  |
| How often visit a park or square | | |  |  |  |  |
|  | At least once a week | |  | 30277 | 48,40% |  |
|  | Less than once a week | |  | 32230 | 51,60% |  |
|  |  |  |  |  |  |  |
| Allergy to plants or insects | | |  |  |  |  |
|  | No |  |  | 41771 | 66,80% |  |
|  | Yes |  |  | 20650 | 33,10% |  |
|  |  |  |  |  |  |  |
| Country |  |  |  |  |  |  |
|  | Chile |  |  | 59157 | 96,70% |  |
|  | Other |  |  | 2030 | 3,30% |  |
|  |  |  |  |  |  |  |
| Education level | |  |  |  |  |  |
|  | Basic |  |  | 1132 | 1,80% |  |
|  | Secondary |  |  | 32859 | 52,60% |  |
|  | Technical |  |  | 12026 | 19,20% |  |
|  | Professional | |  | 13877 | 22,20% |  |
|  | Master / Doctorate | |  | 2613 | 4,20% |  |

Table A2. The impact of green coverage on the positive and negative affect indices

| **Green Coverage %** | **Positive Affect** | | **Negative Affect** | |
| --- | --- | --- | --- | --- |
|  | 1 | 2 | 3 | 4 |
| 8% | 0.153** | 0.161** | -0.425*** | -0.414*** |
|  | (0.0698) | (0.0693) | (0.102) | (0.100) |
| 16% | 0.613*** | 0.651*** | -0.723*** | -0.742*** |
|  | (0.0716) | (0.0718) | (0.0988) | (0.0969) |
| 24% | 0.954*** | 0.946*** | -0.997*** | -0.987*** |
|  | (0.0709) | (0.0705) | (0.0951) | (0.0931) |
| 32% | 1.142*** | 1.193*** | -1.087*** | -1.063*** |
|  | (0.0727) | (0.0717) | (0.101) | (0.0990) |
| 40% | 1.381*** | 1.411*** | -1.156*** | -1.154*** |
|  | (0.0730) | (0.0721) | (0.1000) | (0.0971) |
| 48% | 1.609*** | 1.616*** | -1.315*** | -1.318*** |
|  | (0.0737) | (0.0726) | (0.104) | (0.101) |
| 56% | 1.726*** | 1.733*** | -1.384*** | -1.380*** |
|  | (0.0756) | (0.0746) | (0.103) | (0.101) |
| 64% | 1.880*** | 1.908*** | -1.361*** | -1.357*** |
|  | (0.0788) | (0.0779) | (0.106) | (0.104) |
| 72% | 1.992*** | 2.008*** | -1.422*** | -1.421*** |
|  | (0.0786) | (0.0777) | (0.105) | (0.104) |
| **Constant (0%)** | 4.268*** | 3.708*** | 4.798*** | 5.448*** |
|  | (0.0623) | (0.253) | (0.0922) | (0.445) |
|  |  |  |  |  |
| Controls | No | Yes | No | Yes |
| Observations | 20,786 | 20,786 | 9,716 | 9,716 |
| Number of groups | 2,335 | 2,335 | 1,105 | 1,105 |
| Robust standard errors in parentheses | | | | |
| *** p<0.01, ** p<0.05, * p<0.1 | | | | |

Table A3. Green coverage and positive affect measures, Part 1.

|  | **Interested** | | **Excited** | | **Strong** | | **Inspired** | | **Alert** | |
| --- | --- | --- | --- | --- | --- | --- | --- | --- | --- | --- |
| VARIABLES | 1 | 2 | 3 | 4 | 5 | 6 | 7 | 8 | 9 | 10 |
|  |  |  |  |  |  |  |  |  |  |  |
| Green coverage (+8%) | 0.303*** | 0.296*** | 0.317*** | 0.307*** | 0.151*** | 0.155*** | 0.300*** | 0.296*** | -0.0364** | -0.0406** |
|  | (0.0192) | (0.0184) | (0.0197) | (0.0193) | (0.0207) | (0.0190) | (0.0190) | (0.0185) | (0.0184) | (0.0175) |
| Constant | 3.509*** | 4.627*** | 3.358*** | 3.354*** | 4.904*** | 3.719*** | 4.035*** | 3.517*** | 5.102*** | 5.468*** |
|  | (0.137) | (1.141) | (0.142) | (0.534) | (0.153) | (0.815) | (0.149) | (0.719) | (0.148) | (0.618) |
|  |  |  |  |  |  |  |  |  |  |  |
| Controls | No | Yes | No | Yes | No | Yes | No | Yes | No | Yes |
| Observations | 2,305 | 2,305 | 1,988 | 1,988 | 2,086 | 2,086 | 2,207 | 2,207 | 1,909 | 1,909 |
| Number of groups | 258 | 258 | 224 | 224 | 234 | 234 | 246 | 246 | 214 | 214 |
| Robust standard errors in parentheses | | | | | | | | | | |
| *** p<0.01, ** p<0.05, * p<0.1 | | | | | | | | | | |

Table A4. Green coverage and positive affect measures, Part 2.

|  | **Enthusiastic** | | **Determined** | | **Attentive** | | **Active** | | **Proud** | |
| --- | --- | --- | --- | --- | --- | --- | --- | --- | --- | --- |
| VARIABLES | 11 | 12 | 13 | 14 | 15 | 16 | 17 | 18 | 19 | 20 |
|  |  |  |  |  |  |  |  |  |  |  |
| Green coverage (+8%) | 0.357*** | 0.363*** | 0.186*** | 0.198*** | 0.0967*** | 0.0963*** | 0.270*** | 0.268*** | 0.319*** | 0.321*** |
|  | (0.0201) | (0.0196) | (0.0198) | (0.0200) | (0.0219) | (0.0221) | (0.0214) | (0.0204) | (0.0218) | (0.0212) |
| Constant | 3.193*** | 3.822*** | 5.119*** | 3.908*** | 5.237*** | 6.041*** | 3.559*** | 3.508*** | 3.607*** | 3.121*** |
|  | (0.148) | (0.380) | (0.149) | (0.556) | (0.150) | (0.552) | (0.147) | (0.780) | (0.149) | (0.624) |
|  |  |  |  |  |  |  |  |  |  |  |
| Controls | No | Yes | No | Yes | No | Yes | No | Yes | No | Yes |
| Observations | 2,097 | 2,097 | 1,983 | 1,983 | 2,132 | 2,132 | 2,055 | 2,055 | 2,024 | 2,024 |
| Number of groups | 233 | 233 | 226 | 226 | 242 | 242 | 232 | 232 | 226 | 226 |
| Robust standard errors in parentheses | | | | | | | | | | |
| *** p<0.01, ** p<0.05, * p<0.1 | | | | | | | | | | |

Table A5. Green coverage distribution and positive affect measures, Part 1.

| **Green Coverage %** | **Interested** | | **Excited** | | **Strong** | | **Inspired** | | **Alert** | |
| --- | --- | --- | --- | --- | --- | --- | --- | --- | --- | --- |
|  | 1 | 2 | 3 | 4 | 5 | 6 | 7 | 8 | 9 | 10 |
| 8% | 0.612*** | 0.508*** | 0.111 | 0.0271 | -0.000134 | 0.122 | 0.585*** | 0.529** | -0.252 | -0.405* |
|  | (0.191) | (0.186) | (0.187) | (0.179) | (0.219) | (0.215) | (0.221) | (0.213) | (0.220) | (0.208) |
| 16% | 0.647*** | 0.696*** | 0.697*** | 0.650*** | 0.570*** | 0.690*** | 1.244*** | 1.292*** | 0.108 | -0.0306 |
|  | (0.204) | (0.191) | (0.213) | (0.195) | (0.214) | (0.215) | (0.214) | (0.201) | (0.231) | (0.218) |
| 24% | 1.619*** | 1.522*** | 1.339*** | 1.159*** | 0.403* | 0.510** | 1.774*** | 1.703*** | -0.217 | -0.346* |
|  | (0.209) | (0.193) | (0.190) | (0.184) | (0.216) | (0.218) | (0.224) | (0.216) | (0.215) | (0.200) |
| 32% | 1.614*** | 1.612*** | 1.470*** | 1.434*** | 1.084*** | 1.179*** | 1.802*** | 1.813*** | -0.639*** | -0.727*** |
|  | (0.212) | (0.192) | (0.206) | (0.203) | (0.232) | (0.221) | (0.219) | (0.216) | (0.214) | (0.208) |
| 40% | 1.917*** | 1.940*** | 1.768*** | 1.643*** | 0.757*** | 0.931*** | 2.087*** | 2.037*** | -0.216 | -0.348 |
|  | (0.200) | (0.190) | (0.215) | (0.203) | (0.223) | (0.214) | (0.200) | (0.194) | (0.234) | (0.225) |
| 48% | 2.257*** | 2.133*** | 2.181*** | 1.982*** | 1.066*** | 1.139*** | 2.166*** | 2.160*** | -0.452** | -0.576*** |
|  | (0.194) | (0.184) | (0.224) | (0.205) | (0.241) | (0.224) | (0.224) | (0.217) | (0.229) | (0.211) |
| 56% | 2.414*** | 2.324*** | 2.503*** | 2.292*** | 1.195*** | 1.299*** | 2.621*** | 2.622*** | -0.216 | -0.323 |
|  | (0.215) | (0.200) | (0.206) | (0.192) | (0.230) | (0.223) | (0.215) | (0.212) | (0.226) | (0.219) |
| 64% | 2.664*** | 2.549*** | 2.489*** | 2.465*** | 1.375*** | 1.510*** | 2.691*** | 2.665*** | -0.231 | -0.380 |
|  | (0.222) | (0.204) | (0.227) | (0.219) | (0.270) | (0.259) | (0.219) | (0.212) | (0.244) | (0.246) |
| 72% | 2.778*** | 2.729*** | 2.629*** | 2.527*** | 1.190*** | 1.285*** | 2.893*** | 2.813*** | -0.487** | -0.583*** |
|  | (0.216) | (0.205) | (0.216) | (0.213) | (0.239) | (0.227) | (0.229) | (0.223) | (0.226) | (0.212) |
| **Constant (0%)** | 3.517*** | 4.604*** | 3.575*** | 3.594*** | 4.977*** | 3.785*** | 3.903*** | 3.347*** | 5.165*** | 5.640*** |
|  | (0.167) | (1.177) | (0.169) | (0.534) | (0.202) | (0.808) | (0.191) | (0.735) | (0.195) | (0.615) |
|  |  |  |  |  |  |  |  |  |  |  |
| Controls | No | Yes | No | Yes | No | Yes | No | Yes | No | Yes |
| Observations | 2,305 | 2,305 | 1,988 | 1,988 | 2,086 | 2,086 | 2,207 | 2,207 | 1,909 | 1,909 |
| Number of groups | 258 | 258 | 224 | 224 | 234 | 234 | 246 | 246 | 214 | 214 |
| Robust standard errors in parentheses | | | | | | | | | | |
| *** p<0.01, ** p<0.05, * p<0.1 | | | | | | | | | | |

Table A6. Green coverage distribution and positive affect measures, Part 2.

| **Green Coverage %** | **Enthusiastic** | | **Determined** | | **Attentive** | | **Active** | | **Proud** | | |
| --- | --- | --- | --- | --- | --- | --- | --- | --- | --- | --- | --- |
|  | 11 | 12 | 13 | 14 | 15 | 16 | 17 | 18 | 19 | 20 | |
| 8% | -0.0107 | 0.0729 | 0.209 | 0.282 | -0.446** | -0.445** | 0.230 | 0.189 | 0.307 | 0.347 | |
|  | (0.214) | (0.213) | (0.242) | (0.231) | (0.220) | (0.220) | (0.192) | (0.181) | (0.209) | (0.217) | |
| 16% | 0.895*** | 0.954*** | 0.434** | 0.472** | -0.0978 | -0.0789 | 0.709*** | 0.703*** | 0.765*** | 0.856*** | |
|  | (0.206) | (0.203) | (0.219) | (0.206) | (0.250) | (0.249) | (0.207) | (0.202) | (0.227) | (0.229) | |
| 24% | 1.275*** | 1.280*** | 1.026*** | 0.966*** | -0.0625 | -0.0328 | 0.967*** | 0.909*** | 1.218*** | 1.308*** | |
|  | (0.196) | (0.193) | (0.231) | (0.218) | (0.210) | (0.211) | (0.214) | (0.203) | (0.241) | (0.251) | |
| 32% | 1.623*** | 1.695*** | 0.912*** | 1.047*** | 0.291 | 0.330 | 1.332*** | 1.331*** | 1.729*** | 1.893*** | |
|  | (0.208) | (0.207) | (0.249) | (0.227) | (0.227) | (0.229) | (0.199) | (0.188) | (0.232) | (0.235) | |
| 40% | 2.095*** | 2.130*** | 1.122*** | 1.263*** | 0.391 | 0.433* | 1.715*** | 1.674*** | 2.024*** | 2.065*** | |
|  | (0.207) | (0.201) | (0.214) | (0.206) | (0.249) | (0.250) | (0.214) | (0.198) | (0.245) | (0.241) | |
| 48% | 2.195*** | 2.266*** | 1.602*** | 1.670*** | 0.654*** | 0.654*** | 1.803*** | 1.817*** | 2.346*** | 2.342*** | |
|  | (0.230) | (0.230) | (0.221) | (0.207) | (0.216) | (0.217) | (0.214) | (0.197) | (0.239) | (0.240) | |
| 56% | 2.399*** | 2.542*** | 1.334*** | 1.372*** | 0.424* | 0.431* | 2.027*** | 1.945*** | 2.254*** | 2.375*** | |
|  | (0.213) | (0.210) | (0.237) | (0.230) | (0.223) | (0.224) | (0.224) | (0.208) | (0.260) | (0.270) | |
| 64% | 2.886*** | 2.953*** | 1.494*** | 1.607*** | 0.297 | 0.285 | 2.236*** | 2.176*** | 2.633*** | 2.659*** | |
|  | (0.213) | (0.216) | (0.241) | (0.235) | (0.233) | (0.233) | (0.238) | (0.223) | (0.237) | (0.245) | |
| 72% | 3.093*** | 3.159*** | 1.678*** | 1.835*** | 0.682*** | 0.697*** | 2.311*** | 2.325*** | 2.793*** | 2.882*** | |
|  | (0.228) | (0.225) | (0.243) | (0.245) | (0.250) | (0.252) | (0.232) | (0.223) | (0.255) | (0.255) | |
| **Constant (0%)** | 3.514*** | 4.094*** | 5.150*** | 3.972*** | 5.552*** | 6.357*** | 3.710*** | 3.669*** | 3.746*** | 3.229*** | |
|  | (0.181) | (0.397) | (0.196) | (0.562) | (0.187) | (0.564) | (0.173) | (0.785) | (0.198) | (0.672) | |
|  |  |  |  |  |  |  |  |  |  |  | |
| Controls | No | Yes | No | Yes | No | Yes | No | Yes | No | Yes | |
| Observations | 2,097 | 2,097 | 1,983 | 1,983 | 2,132 | 2,132 | 2,055 | 2,055 | 2,024 | 2,024 | |
| Number of groups | 233 | 233 | 226 | 226 | 242 | 242 | 232 | 232 | 226 | 226 | |
| Robust standard errors in parentheses | | | | | | | | | | |  |
| *** p<0.01, ** p<0.05, * p<0.1 | | | | | | | | | | |  |

Table A7. Green coverage and negative affect measures, Part 1.

|  | **Distressed** | | **Upset** | | **Guilty** | | **Scared** | | **Hostile** | |
| --- | --- | --- | --- | --- | --- | --- | --- | --- | --- | --- |
| VARIABLES | 1 | 2 | 3 | 4 | 5 | 6 | 7 | 8 | 9 | 10 |
|  |  |  |  |  |  |  |  |  |  |  |
| Green coverage (+8%) | -0.160*** | -0.162*** | -0.232*** | -0.228*** | -0.0851*** | -0.0844*** | -0.0702*** | -0.0656*** | -0.149*** | -0.153*** |
|  | (0.0326) | (0.0329) | (0.0290) | (0.0276) | (0.0291) | (0.0281) | (0.0242) | (0.0227) | (0.0250) | (0.0257) |
| Constant | 4.977*** | 3.862*** | 4.908*** | 5.071*** | 4.022*** | 7.226*** | 4.001*** | 5.197*** | 4.904*** | 5.330*** |
|  | (0.228) | (1.089) | (0.247) | (0.970) | (0.256) | (0.574) | (0.198) | (0.0681) | (0.217) | (1.194) |
|  |  |  |  |  |  |  |  |  |  |  |
| Controls | No | Yes | No | Yes | No | Yes | No | Yes | No | Yes |
| Observations | 908 | 908 | 1,007 | 1,007 | 985 | 985 | 925 | 925 | 1,007 | 1,007 |
| Number of groups | 104 | 104 | 114 | 114 | 112 | 112 | 108 | 108 | 116 | 116 |
| Robust standard errors in parentheses | | | | | | | | | | |
| *** p<0.01, ** p<0.05, * p<0.1 | | | | | | | | | | |

Table A8. Green coverage and negative affect measures, Part 2.

|  | **Irritable** | | **Nervous** | | **Ashamed** | | **Jittery** | | **Afraid** | |
| --- | --- | --- | --- | --- | --- | --- | --- | --- | --- | --- |
| VARIABLES | 11 | 12 | 13 | 14 | 15 | 16 | 17 | 18 | 19 | 20 |
|  |  |  |  |  |  |  |  |  |  |  |
| Green coverage (+8%) | -0.256*** | -0.259*** | -0.107*** | -0.104*** | -0.147*** | -0.147*** | -0.128*** | -0.126*** | -0.0745*** | -0.0816*** |
|  | (0.0366) | (0.0365) | (0.0259) | (0.0262) | (0.0275) | (0.0278) | (0.0242) | (0.0225) | (0.0247) | (0.0229) |
| Constant | 5.336*** | 5.488*** | 5.051*** | 6.181*** | 3.758*** | 4.718*** | 4.546*** | 3.083*** | 4.364*** | 4.370*** |
|  | (0.251) | (0.509) | (0.195) | (1.012) | (0.255) | (1.040) | (0.220) | (0.353) | (0.227) | (0.805) |
|  |  |  |  |  |  |  |  |  |  |  |
| Controls | No | Yes | No | Yes | No | Yes | No | Yes | No | Yes |
| Observations | 1,031 | 1,031 | 1,015 | 1,015 | 990 | 990 | 1,071 | 1,071 | 777 | 777 |
| Number of groups | 115 | 115 | 115 | 115 | 111 | 111 | 122 | 122 | 88 | 88 |
| Robust standard errors in parentheses | | | | | | | | | | |
| *** p<0.01, ** p<0.05, * p<0.1 | | | | | | | | | | |

Table A9. Green coverage distribution and negative affect measures, Part 1.

| **Green Coverage %** | **Distressed** | | **Upset** | | **Guilty** | | **Scared** | | **Hostile** | |
| --- | --- | --- | --- | --- | --- | --- | --- | --- | --- | --- |
|  | 1 | 2 | 3 | 4 | 5 | 6 | 7 | 8 | 9 | 10 |
| 8% | -0.200 | -0.0835 | -0.766** | -0.704** | -0.697* | -0.634* | -0.412 | -0.451* | -0.386 | -0.493 |
|  | (0.348) | (0.359) | (0.330) | (0.340) | (0.365) | (0.362) | (0.271) | (0.244) | (0.333) | (0.317) |
| 16% | -0.697* | -0.711* | -1.193*** | -1.216*** | -0.807** | -0.823** | -0.425* | -0.528** | -0.144 | -0.267 |
|  | (0.384) | (0.388) | (0.281) | (0.276) | (0.354) | (0.358) | (0.241) | (0.212) | (0.352) | (0.351) |
| 24% | -0.917*** | -0.859** | -1.416*** | -1.445*** | -1.000*** | -0.965*** | -0.686*** | -0.626*** | -1.100*** | -1.062*** |
|  | (0.355) | (0.355) | (0.261) | (0.260) | (0.295) | (0.287) | (0.235) | (0.211) | (0.315) | (0.313) |
| 32% | -1.199*** | -1.192*** | -1.843*** | -1.707*** | -0.970*** | -0.885*** | -0.600** | -0.627** | -0.781*** | -0.870*** |
|  | (0.378) | (0.378) | (0.305) | (0.305) | (0.335) | (0.324) | (0.266) | (0.257) | (0.292) | (0.298) |
| 40% | -1.108*** | -1.065*** | -1.824*** | -1.663*** | -1.109*** | -1.056*** | -0.566** | -0.568** | -0.852*** | -0.933*** |
|  | (0.382) | (0.383) | (0.320) | (0.309) | (0.309) | (0.304) | (0.283) | (0.259) | (0.311) | (0.309) |
| 48% | -1.599*** | -1.613*** | -2.010*** | -1.986*** | -1.056*** | -1.013*** | -0.595** | -0.542** | -1.201*** | -1.259*** |
|  | (0.390) | (0.388) | (0.290) | (0.291) | (0.343) | (0.330) | (0.282) | (0.260) | (0.337) | (0.331) |
| 56% | -1.506*** | -1.553*** | -2.198*** | -2.223*** | -1.175*** | -1.155*** | -0.800*** | -0.794*** | -1.221*** | -1.264*** |
|  | (0.372) | (0.372) | (0.306) | (0.295) | (0.346) | (0.331) | (0.290) | (0.262) | (0.324) | (0.319) |
| 64% | -1.304*** | -1.280*** | -2.388*** | -2.246*** | -0.998*** | -0.960*** | -0.783*** | -0.801*** | -1.454*** | -1.489*** |
|  | (0.375) | (0.369) | (0.334) | (0.320) | (0.345) | (0.329) | (0.280) | (0.248) | (0.315) | (0.325) |
| 72% | -1.405*** | -1.312*** | -2.187*** | -2.207*** | -1.098*** | -1.083*** | -0.817*** | -0.805*** | -1.337*** | -1.472*** |
|  | (0.425) | (0.415) | (0.282) | (0.278) | (0.320) | (0.314) | (0.306) | (0.293) | (0.294) | (0.313) |
| **Constant (0%)** | 5.088*** | 3.807*** | 5.224*** | 5.313*** | 4.441*** | 7.633*** | 4.183*** | 5.528*** | 4.940*** | 5.527*** |
|  | (0.307) | (1.096) | (0.290) | (0.970) | (0.310) | (0.593) | (0.246) | (0.212) | (0.282) | (1.134) |
|  |  |  |  |  |  |  |  |  |  |  |
| Controls | No | Yes | No | Yes | No | Yes | No | Yes | No | Yes |
| Observations | 908 | 908 | 1,007 | 1,007 | 985 | 985 | 925 | 925 | 1,007 | 1,007 |
| Number of groups | 104 | 104 | 114 | 114 | 112 | 112 | 108 | 108 | 116 | 116 |
| Robust standard errors in parentheses | | | | | | | | | | |
| *** p<0.01, ** p<0.05, * p<0.1 | | | | | | | | | | |

Table A10. Green coverage distribution and negative affect measures, Part 2.

| **Green Coverage %** | **Irritable** | | **Nervous** | | **Ashamed** | | **Jittery** | | **Afraid** | |  |
| --- | --- | --- | --- | --- | --- | --- | --- | --- | --- | --- | --- |
|  | 11 | 12 | 13 | 14 | 15 | 16 | 17 | 18 | 19 | 20 |  |
| 8% | -0.0212 | 0.0227 | -0.523 | -0.474 | -0.671** | -0.701*** | -0.386 | -0.624** | -0.442 | -0.280 |  |
|  | (0.328) | (0.326) | (0.321) | (0.308) | (0.262) | (0.264) | (0.297) | (0.277) | (0.352) | (0.298) |  |
| 16% | -0.837** | -0.811** | -0.716** | -0.707** | -0.976*** | -0.960*** | -0.842*** | -0.958*** | -0.643*** | -0.618*** |  |
|  | (0.349) | (0.353) | (0.288) | (0.277) | (0.274) | (0.274) | (0.312) | (0.279) | (0.246) | (0.209) |  |
| 24% | -1.167*** | -1.145*** | -0.861*** | -0.825*** | -0.964*** | -0.974*** | -1.016*** | -1.143*** | -0.754*** | -0.877*** |  |
|  | (0.382) | (0.382) | (0.279) | (0.275) | (0.254) | (0.256) | (0.291) | (0.270) | (0.287) | (0.252) |  |
| 32% | -1.421*** | -1.410*** | -1.057*** | -0.979*** | -0.948*** | -0.949*** | -1.461*** | -1.529*** | -0.401 | -0.442 |  |
|  | (0.363) | (0.361) | (0.287) | (0.280) | (0.270) | (0.274) | (0.309) | (0.295) | (0.329) | (0.289) |  |
| 40% | -1.509*** | -1.530*** | -0.966*** | -0.929*** | -1.389*** | -1.390*** | -1.102*** | -1.277*** | -1.081*** | -1.079*** |  |
|  | (0.368) | (0.367) | (0.327) | (0.312) | (0.285) | (0.286) | (0.256) | (0.233) | (0.291) | (0.271) |  |
| 48% | -1.937*** | -1.929*** | -1.062*** | -1.179*** | -1.413*** | -1.378*** | -1.479*** | -1.549*** | -0.602* | -0.756** |  |
|  | (0.391) | (0.390) | (0.321) | (0.324) | (0.272) | (0.270) | (0.280) | (0.257) | (0.353) | (0.307) |  |
| 56% | -2.076*** | -2.043*** | -1.015*** | -0.970*** | -1.416*** | -1.428*** | -1.261*** | -1.303*** | -1.023*** | -0.949*** |  |
|  | (0.393) | (0.392) | (0.273) | (0.275) | (0.326) | (0.324) | (0.273) | (0.254) | (0.301) | (0.280) |  |
| 64% | -2.021*** | -2.037*** | -1.015*** | -1.017*** | -1.376*** | -1.390*** | -1.243*** | -1.330*** | -0.754*** | -0.867*** |  |
|  | (0.415) | (0.416) | (0.324) | (0.316) | (0.281) | (0.284) | (0.309) | (0.293) | (0.285) | (0.277) |  |
| 72% | -2.107*** | -2.106*** | -1.320*** | -1.219*** | -1.691*** | -1.696*** | -1.323*** | -1.423*** | -0.906*** | -0.847*** |  |
|  | (0.400) | (0.403) | (0.307) | (0.308) | (0.348) | (0.351) | (0.273) | (0.249) | (0.347) | (0.317) |  |
| **Constant (0%)** | 5.234*** | 5.355*** | 5.317*** | 6.455*** | 4.022*** | 4.965*** | 4.845*** | 3.260*** | 4.614*** | 4.656*** |  |
|  | (0.332) | (0.544) | (0.255) | (0.994) | (0.306) | (1.055) | (0.267) | (0.363) | (0.282) | (0.781) |  |
|  |  |  |  |  |  |  |  |  |  |  |  |
| Controls | No | Yes | No | Yes | No | Yes | No | Yes | No | Yes |  |
| Observations | 1,031 | 1,031 | 1,015 | 1,015 | 990 | 990 | 1,071 | 1,071 | 777 | 777 |  |
| Number of groups | 115 | 115 | 115 | 115 | 111 | 111 | 122 | 122 | 88 | 88 |  |
| Robust standard errors in parentheses | | | | | | | | | | | |
| *** p<0.01, ** p<0.05, * p<0.1 | | | | | | | | | | | |

Table A11. Green coverage and PA by neighbourhood income level

|  | **Positive Emotions by Income Level** | | | | | |
| --- | --- | --- | --- | --- | --- | --- |
|  | Low Income | | Middle Income | | High Income | |
| VARIABLES | 1 | 2 | 3 | 4 | 5 | 6 |
|  |  |  |  |  |  |  |
| Green coverage (+8%) | 0.212*** | 0.212*** | 0.199*** | 0.199*** | 0.271*** | 0.272*** |
|  | (0.00933) | (0.00938) | (0.00917) | (0.00923) | (0.0102) | (0.0102) |
|  |  |  |  |  |  |  |
| Controls | No | Yes | No | Yes | No | Yes |
| Constant | 3.731*** | 3.995*** | 4.194*** | 4.768*** | 4.575*** | 4.816*** |
|  | (0.0660) | (0.285) | (0.0631) | (0.295) | (0.0728) | (0.350) |
|  |  |  |  |  |  |  |
| Observations | 6,929 | 6,929 | 6,932 | 6,932 | 6,925 | 6,925 |
| Number of groups | 2,322 | 2,322 | 2,327 | 2,327 | 2,321 | 2,321 |
| Robust standard errors in parentheses | | | | | | |
| *** p<0.01, ** p<0.05, * p<0.1 | | | | | | |

Table A12. Green coverage and NA by neighbourhood income level

|  | **Negative emotions by Income Level** | | | | | |
| --- | --- | --- | --- | --- | --- | --- |
|  | Low Income | | Middle Income | | High Income | |
| VARIABLES | 1 | 2 | 3 | 4 | 5 | 6 |
|  |  |  |  |  |  |  |
| Green coverage (+8%) | -0.141*** | -0.141*** | -0.139*** | -0.140*** | -0.137*** | -0.137*** |
|  | (0.0132) | (0.0132) | (0.0127) | (0.0127) | (0.0139) | (0.0139) |
|  |  |  |  |  |  |  |
| Controls | No | Yes | No | Yes | No | Yes |
| Constant | 5.089*** | 4.537*** | 4.636*** | 4.879*** | 3.993*** | 4.881*** |
|  | (0.0982) | (0.511) | (0.0944) | (0.506) | (0.105) | (0.577) |
|  |  |  |  |  |  |  |
| Observations | 3,241 | 3,241 | 3,238 | 3,238 | 3,237 | 3,237 |
| Number of groups | 1,097 | 1,097 | 1,093 | 1,093 | 1,090 | 1,090 |
| Robust standard errors in parentheses | | | | | | |
| *** p<0.01, ** p<0.05, * p<0.1 | | | | | | |

Table A13. Green coverage distribution and PA by neighbourhood income level

|  | **Positive Emotions by Income level** | | | | | |
| --- | --- | --- | --- | --- | --- | --- |
| **Green Coverage %** | **Low Income** | | **Medium Income** | | **High Income** | |
|  | 1 | 2 | 3 | 4 | 5 | 6 |
| 8% | 0.168 | 0.188* | 0.0335 | 0.00427 | 0.192* | 0.227* |
|  | (0.112) | (0.112) | (0.109) | (0.109) | (0.116) | (0.117) |
| 16% | 0.665*** | 0.669*** | 0.156 | 0.160 | 0.931*** | 0.966*** |
|  | (0.112) | (0.112) | (0.108) | (0.109) | (0.118) | (0.120) |
| 24% | 1.061*** | 1.072*** | 0.527*** | 0.509*** | 1.151*** | 1.179*** |
|  | (0.109) | (0.110) | (0.103) | (0.104) | (0.115) | (0.116) |
| 32% | 1.088*** | 1.098*** | 0.852*** | 0.875*** | 1.571*** | 1.618*** |
|  | (0.110) | (0.111) | (0.113) | (0.113) | (0.120) | (0.121) |
| 40% | 1.296*** | 1.320*** | 1.151*** | 1.136*** | 1.636*** | 1.681*** |
|  | (0.111) | (0.112) | (0.107) | (0.108) | (0.117) | (0.118) |
| 48% | 1.402*** | 1.415*** | 1.394*** | 1.393*** | 1.874*** | 1.910*** |
|  | (0.116) | (0.117) | (0.109) | (0.110) | (0.115) | (0.117) |
| 56% | 1.618*** | 1.624*** | 1.339*** | 1.333*** | 2.081*** | 2.119*** |
|  | (0.116) | (0.117) | (0.110) | (0.111) | (0.119) | (0.121) |
| 64% | 1.817*** | 1.822*** | 1.482*** | 1.478*** | 2.321*** | 2.348*** |
|  | (0.118) | (0.119) | (0.110) | (0.110) | (0.127) | (0.127) |
| 72% | 1.922*** | 1.941*** | 1.539*** | 1.532*** | 2.396*** | 2.433*** |
|  | (0.118) | (0.119) | (0.110) | (0.111) | (0.125) | (0.126) |
| **Constant (0%)** | 3.791*** | 4.031*** | 4.439*** | 5.031*** | 4.651*** | 4.821*** |
|  | (0.0932) | (0.294) | (0.0825) | (0.301) | (0.0982) | (0.359) |
|  |  |  |  |  |  |  |
| Controls | No | Yes | No | Yes | No | Yes |
| Observations | 6,929 | 6,929 | 6,932 | 6,932 | 6,925 | 6,925 |
| Number of groups | 2,322 | 2,322 | 2,327 | 2,327 | 2,321 | 2,321 |
| Robust standard errors in parentheses | | | | | | |
| *** p<0.01, ** p<0.05, * p<0.1 | | | | | | |

Table A14. Green coverage distribution and NA by neighbourhood income level

|  | **Negative Emotions by Income Level** | | | | | |
| --- | --- | --- | --- | --- | --- | --- |
| **Green Coverage %** | **Low Income** | | **Medium Income** | | **High Income** | |
|  | 1 | 2 | 3 | 4 | 5 | 6 |
| 8% | -0.609*** | -0.637*** | -0.0985 | -0.0887 | -0.250 | -0.247 |
|  | (0.162) | (0.162) | (0.167) | (0.167) | (0.177) | (0.177) |
| 16% | -0.866*** | -0.882*** | -0.368** | -0.368** | -0.803*** | -0.801*** |
|  | (0.163) | (0.163) | (0.156) | (0.156) | (0.163) | (0.163) |
| 24% | -1.333*** | -1.336*** | -0.530*** | -0.541*** | -1.003*** | -0.990*** |
|  | (0.148) | (0.148) | (0.157) | (0.157) | (0.158) | (0.158) |
| 32% | -1.088*** | -1.100*** | -0.847*** | -0.848*** | -1.000*** | -0.994*** |
|  | (0.155) | (0.155) | (0.157) | (0.157) | (0.157) | (0.157) |
| 40% | -1.230*** | -1.248*** | -0.817*** | -0.813*** | -1.184*** | -1.179*** |
|  | (0.156) | (0.156) | (0.153) | (0.153) | (0.169) | (0.169) |
| 48% | -1.370*** | -1.386*** | -1.242*** | -1.238*** | -1.167*** | -1.166*** |
|  | (0.163) | (0.163) | (0.158) | (0.158) | (0.162) | (0.162) |
| 56% | -1.549*** | -1.557*** | -1.147*** | -1.150*** | -1.360*** | -1.361*** |
|  | (0.158) | (0.158) | (0.149) | (0.149) | (0.168) | (0.168) |
| 64% | -1.424*** | -1.444*** | -1.094*** | -1.098*** | -1.402*** | -1.390*** |
|  | (0.166) | (0.166) | (0.162) | (0.162) | (0.169) | (0.169) |
| 72% | -1.564*** | -1.574*** | -1.138*** | -1.137*** | -1.267*** | -1.264*** |
|  | (0.164) | (0.165) | (0.163) | (0.163) | (0.175) | (0.175) |
| **Constant (0%)** | 5.417*** | 4.866*** | 4.595*** | 4.846*** | 4.179*** | 5.046*** |
|  | (0.133) | (0.523) | (0.129) | (0.516) | (0.145) | (0.579) |
|  |  |  |  |  |  |  |
| Controls | No | Yes | No | Yes | No | Yes |
| Observations | 3,241 | 3,241 | 3,238 | 3,238 | 3,237 | 3,237 |
| Number of groups | 1,097 | 1,097 | 1,093 | 1,093 | 1,090 | 1,090 |
| Robust standard errors in parentheses | | | | | | |
| *** p<0.01, ** p<0.05, * p<0.1 | | | | | | |
